# Separation of base allele and sampling term effects gives new insights in variance component QTL analysis

**DOI:** 10.1186/1471-2156-8-1

**Published:** 2007-01-08

**Authors:** Lars Rönnegård, Örjan Carlborg

**Affiliations:** 1 Linnaeus Centre for Bioinformatics, Uppsala University, BMC, Box 598, SE-75124 Uppsala, Sweden

## Abstract

**Background:**

Variance component (VC) models are commonly used for Quantitative Trait Loci (QTL) mapping in outbred populations. Here, the QTL effect is given as a random effect and a critical part of the model is the relationship between the phenotypic values and the random effect. In the traditional VC model, each individual has a unique QTL effect and the relationship between these random effects is given as a covariance structure (known as the identity-by-descent (IBD) matrix).

**Results:**

We present an alternative notation of the variance component model, where the elements of the random effect are independent base generation allele effects and sampling term effects. The relationship between the phenotypic vales and the random effect is given by an incidence matrix, which results in a novel, but statistically equivalent, version of the traditional VC model. A general algorithm to estimate this incidence matrix is presented. Since the model is given in terms of base generation allele effects and sampling term effects, these effects can be estimated separately using best linear unbiased prediction (BLUP). From simulated data, we showed that biallelic QTL effects could be accurately clustered using the BLUP obtained from our model notation when markers are fully informative, and that the accuracy increased with the size of the QTL effect. We also developed a measure indicating whether a base generation marker homozygote is a QTL heterozygote or not, by comparing the variances of the sampling term BLUP and the base generation allele BLUP. A ratio greater than one gives strong support for a QTL heterozygote.

**Conclusion:**

We developed a simple presentation of the VC QTL model for identification of base generation allele effects in QTL linkage analysis. The base generation allele effects and sampling term effects were separated in our model notation. This clarifies the assumptions of the model and should also enhance the development of genome scan methods.

## Background

Understanding the genetic architecture of complex traits controlled by many genes and environmental factors is currently one of the grand challenges in genetics. In this quest for the deciphering of the genetic code, Quantitative Trait Loci (QTL) mapping can be a powerful statistical tool. The basic idea of QTL analysis is to trace the inheritance of alleles from founders through a pedigree by using genetic markers. After estimating this gene flow through the pedigree, the allelic effects are estimated by relating the phenotypes with the different alleles. The position in the genome having the greatest statistical evidence for large allelic effects is the most likely position of a QTL.

In QTL studies of pedigrees in outbred populations, variance component (VC) models are commonly used to estimate the variance of the allelic effects [1], rather than the effect of each individual allele. The studied phenotype is the explanatory variable and the QTL effect is assumed to be a random part of the phenotype. It is random because the founders of the mapping population are assumed to have QTL alleles with effects drawn from a distribution of allelic effects in the entire population and also because the alleles are transmitted from ancestor to descendent by a random process. The model assumes that the random effect is sampled from a multivariate normal distribution with an infinite number of different alleles, and the model is therefore called *the infinite alleles model*. Simulations have shown that the model is capable of giving unbiased estimates also when the QTL is biallelic [2-5].

To be able to estimate the variance of the random QTL effect, a between-individual covariance structure has to be specified. For a non-inbred population this is equal to the proportion of genes that individuals share identical-by-descent at a specific position [6], and the matrix describing the covariance structure is therefore called the identity-by-descent (IBD) matrix. Since the IBD matrix is not known *a priori*, it has to be estimated from marker information. This matrix has applications beyond the VC QTL model [7,8], however, and a lot of effort has been put into developing IBD estimation algorithms. For small pedigrees and few markers, the most likely IBD matrix can be estimated [9], but for large pedigrees this is too computationally demanding and approximate algorithms have therefore also been developed [10-12]. Hence, the IBD matrix estimation algorithms have been described in detail, but explicit definitions of the random QTL effect in terms of independent levels are more difficult to find (see however [13,14]).

Several review articles have been published where the assumptions of the model are addressed [2,15-18]. The focus of these papers was on statistical testing of QTL effects and interpretation of genome scans. Several other papers have focused on the biological interpretation of the random QTL effects. Goddard [14] compares the assumptions of the biallelic and the infinite alleles model. He also explains how uncertainty is included in the infinite alleles model by adding a random sampling term to the expected allelic effects and that the variance of the sampling terms are proportional to the QTL variance under the infinite alleles model. Furthermore, Meuwissen and Goddard [19] developed a VC model where they related phenotypes with QTL allele effects by means of an incidence matrix. None of these papers show, however, how the random QTL effect can be given in terms of independent levels. Such a development would more clearly show the definitions of the levels in the random QTL effect and thereby help us to interpret the results from the VC QTL model.

The aim of this paper is to develop a simple presentation of the VC QTL model for identification of base generation allele effects in QTL linkage analysis. Although our main objective is to better understand the biological functions of the random QTL effect, we argue that our model formulation may also enhance the development genome scan methods.

## Results

In this section we show how an alternative incidence matrix based VC QTL model is formulated, we give the prerequisites for the model to be equivalent to the IBD matrix based model, and we also present a general algorithm for constructing the incidence matrix. The choice of notation affects the results that can be obtained from the model in terms of best linear unbiased predictions (BLUP) [20]. This is shown with simulations and is also illustrated with an analysis of real data from a wild-domestic chicken cross. The theoretical details are given in the Methods section.

### An incidence matrix based VC QTL model

The alternative incidence matrix notation breaks down the VC model to its most basic form where the levels of the random effect are independent. An advantage of this approach is that it is easy to identify the assumptions directly from the model. The present paper is restricted to VC models where there are additive and dominance effects but the models are easily extended to include polygenic, family specific effects, epistasis and genotype by environmental interactions following the models given in [1,21-23]. A VC model may consist of fixed and random effects (a mixed model) but the main parameter of interest is the variance of the random QTL effect, and the fixed effects are therefore ignored without loss of generality in the presentation below.

A restriction on the random QTL effect **v **can be given either by the covariance structure between individuals, i.e. the IBD matrix **Π **or alternatively by an incidence matrix **Z **relating individuals with the QTL alleles in the base generation. In the latter case, the elements in the vector of random effects v* are independent. The trait vector **y **is multivariate normal and the distribution of the random effects, i.e. the QTL allele effects, is given by *Q *~ MVN(0, 12σv2
 MathType@MTEF@5@5@+=feaafiart1ev1aaatCvAUfKttLearuWrP9MDH5MBPbIqV92AaeXatLxBI9gBaebbnrfifHhDYfgasaacH8akY=wiFfYdH8Gipec8Eeeu0xXdbba9frFj0=OqFfea0dXdd9vqai=hGuQ8kuc9pgc9s8qqaq=dirpe0xb9q8qiLsFr0=vr0=vr0dc8meaabaqaciaacaGaaeqabaqabeGadaaakeaadaWcaaqaaiabigdaXaqaaiabikdaYaaaiiGacqWFdpWCdaqhaaWcbaGaemODayhabaGaeGOmaidaaaaa@32FC@**I**) where **I **is the identity matrix and σv2
 MathType@MTEF@5@5@+=feaafiart1ev1aaatCvAUfKttLearuWrP9MDH5MBPbIqV92AaeXatLxBI9gBaebbnrfifHhDYfgasaacH8akY=wiFfYdH8Gipec8Eeeu0xXdbba9frFj0=OqFfea0dXdd9vqai=hGuQ8kuc9pgc9s8qqaq=dirpe0xb9q8qiLsFr0=vr0=vr0dc8meaabaqaciaacaGaaeqabaqabeGadaaakeaaiiGacqWFdpWCdaqhaaWcbaGaemODayhabaGaeGOmaidaaaaa@310A@ is the QTL genotypic effect. The genotypic value *v*_*i *_of individual *i *in the base generation is the sum of the pair of QTL allele effects at a specific position *v*_*i *_= *Q*_*k *_+ *Q*_*k*+1_, where the QTL alleles are arbitrarily numbered *k *= 2*i*-1 in the base. Hence, by defining the variance of the random QTL genotypic effects as σv2
 MathType@MTEF@5@5@+=feaafiart1ev1aaatCvAUfKttLearuWrP9MDH5MBPbIqV92AaeXatLxBI9gBaebbnrfifHhDYfgasaacH8akY=wiFfYdH8Gipec8Eeeu0xXdbba9frFj0=OqFfea0dXdd9vqai=hGuQ8kuc9pgc9s8qqaq=dirpe0xb9q8qiLsFr0=vr0=vr0dc8meaabaqaciaacaGaaeqabaqabeGadaaakeaaiiGacqWFdpWCdaqhaaWcbaGaemODayhabaGaeGOmaidaaaaa@310A@, the variance of the QTL allele effects is 12σv2
 MathType@MTEF@5@5@+=feaafiart1ev1aaatCvAUfKttLearuWrP9MDH5MBPbIqV92AaeXatLxBI9gBaebbnrfifHhDYfgasaacH8akY=wiFfYdH8Gipec8Eeeu0xXdbba9frFj0=OqFfea0dXdd9vqai=hGuQ8kuc9pgc9s8qqaq=dirpe0xb9q8qiLsFr0=vr0=vr0dc8meaabaqaciaacaGaaeqabaqabeGadaaakeaadaWcaaqaaiabigdaXaqaaiabikdaYaaaiiGacqWFdpWCdaqhaaWcbaGaemODayhabaGaeGOmaidaaaaa@32FC@. The QTL alleles are all assumed to be independent in the base generation, i.e. Cov(*Q*_*i*_, *Q*_*j*_) = 0 where *i *and *j *are different indices for the *m *base alleles. The VC QTL model assumes that all alleles are different in the base generation so that *m *equals twice the number of base generation individuals.

The incidence presentation of the VC QTL model is:

**y **= *μ *+ Zv* + e     (1)

Here *μ *is the overall mean, v* is the vector of *m *independently normally distributed base generation alleles with v* ~ MVN(0,12σv2
 MathType@MTEF@5@5@+=feaafiart1ev1aaatCvAUfKttLearuWrP9MDH5MBPbIqV92AaeXatLxBI9gBaebbnrfifHhDYfgasaacH8akY=wiFfYdH8Gipec8Eeeu0xXdbba9frFj0=OqFfea0dXdd9vqai=hGuQ8kuc9pgc9s8qqaq=dirpe0xb9q8qiLsFr0=vr0=vr0dc8meaabaqaciaacaGaaeqabaqabeGadaaakeaadaWcaaqaaiabigdaXaqaaiabikdaYaaaiiGacqWFdpWCdaqhaaWcbaGaemODayhabaGaeGOmaidaaaaa@32FC@). The matrix **Z **is of size *n *× *m*, **e **is the vector of residuals with **e **~ MVN(0, **I**σe2
 MathType@MTEF@5@5@+=feaafiart1ev1aaatCvAUfKttLearuWrP9MDH5MBPbIqV92AaeXatLxBI9gBaebbnrfifHhDYfgasaacH8akY=wiFfYdH8Gipec8Eeeu0xXdbba9frFj0=OqFfea0dXdd9vqai=hGuQ8kuc9pgc9s8qqaq=dirpe0xb9q8qiLsFr0=vr0=vr0dc8meaabaqaciaacaGaaeqabaqabeGadaaakeaaiiGacqWFdpWCdaqhaaWcbaGaemyzaugabaGaeGOmaidaaaaa@30E8@) where σe2
 MathType@MTEF@5@5@+=feaafiart1ev1aaatCvAUfKttLearuWrP9MDH5MBPbIqV92AaeXatLxBI9gBaebbnrfifHhDYfgasaacH8akY=wiFfYdH8Gipec8Eeeu0xXdbba9frFj0=OqFfea0dXdd9vqai=hGuQ8kuc9pgc9s8qqaq=dirpe0xb9q8qiLsFr0=vr0=vr0dc8meaabaqaciaacaGaaeqabaqabeGadaaakeaaiiGacqWFdpWCdaqhaaWcbaGaemyzaugabaGaeGOmaidaaaaa@30E8@ is the residual variance. The variance of **y **is therefore V=12ZZ′σv2+Iσe2
 MathType@MTEF@5@5@+=feaafiart1ev1aaatCvAUfKttLearuWrP9MDH5MBPbIqV92AaeXatLxBI9gBaebbnrfifHhDYfgasaacH8akY=wiFfYdH8Gipec8Eeeu0xXdbba9frFj0=OqFfea0dXdd9vqai=hGuQ8kuc9pgc9s8qqaq=dirpe0xb9q8qiLsFr0=vr0=vr0dc8meaabaqaciaacaGaaeqabaqabeGadaaakeaaieqacqWFwbGvcqGH9aqpdaWcaaqaaiabigdaXaqaaiabikdaYaaacqWFAbGwcuWFAbGwgaqbaGGaciab+n8aZnaaDaaaleaacqWG2bGDaeaacqaIYaGmaaGccqGHRaWkcqWFjbqscqGFdpWCdaqhaaWcbaGaemyzaugabaGaeGOmaidaaaaa@3DEC@

The traditional IBD matrix version [1] is given by:

**y **= *μ *+ **v + e **    (2)

where **v **is the vector of QTL genotype effects (length *n*) and **v **~ MVN(0, Πσv2
 MathType@MTEF@5@5@+=feaafiart1ev1aaatCvAUfKttLearuWrP9MDH5MBPbIqV92AaeXatLxBI9gBaebbnrfifHhDYfgasaacH8akY=wiFfYdH8Gipec8Eeeu0xXdbba9frFj0=OqFfea0dXdd9vqai=hGuQ8kuc9pgc9s8qqaq=dirpe0xb9q8qiLsFr0=vr0=vr0dc8meaabaqaciaacaGaaeqabaqabeGadaaakeaaiiGacqWFdpWCdaqhaaWcbaGaemODayhabaGaeGOmaidaaaaa@310A@) and **e **~ MVN(0, **I**σe2
 MathType@MTEF@5@5@+=feaafiart1ev1aaatCvAUfKttLearuWrP9MDH5MBPbIqV92AaeXatLxBI9gBaebbnrfifHhDYfgasaacH8akY=wiFfYdH8Gipec8Eeeu0xXdbba9frFj0=OqFfea0dXdd9vqai=hGuQ8kuc9pgc9s8qqaq=dirpe0xb9q8qiLsFr0=vr0=vr0dc8meaabaqaciaacaGaaeqabaqabeGadaaakeaaiiGacqWFdpWCdaqhaaWcbaGaemyzaugabaGaeGOmaidaaaaa@30E8@). Thus the variance of **y **is **V **= **Π**σv2
 MathType@MTEF@5@5@+=feaafiart1ev1aaatCvAUfKttLearuWrP9MDH5MBPbIqV92AaeXatLxBI9gBaebbnrfifHhDYfgasaacH8akY=wiFfYdH8Gipec8Eeeu0xXdbba9frFj0=OqFfea0dXdd9vqai=hGuQ8kuc9pgc9s8qqaq=dirpe0xb9q8qiLsFr0=vr0=vr0dc8meaabaqaciaacaGaaeqabaqabeGadaaakeaaiiGacqWFdpWCdaqhaaWcbaGaemODayhabaGaeGOmaidaaaaa@310A@ + **I**σe2
 MathType@MTEF@5@5@+=feaafiart1ev1aaatCvAUfKttLearuWrP9MDH5MBPbIqV92AaeXatLxBI9gBaebbnrfifHhDYfgasaacH8akY=wiFfYdH8Gipec8Eeeu0xXdbba9frFj0=OqFfea0dXdd9vqai=hGuQ8kuc9pgc9s8qqaq=dirpe0xb9q8qiLsFr0=vr0=vr0dc8meaabaqaciaacaGaaeqabaqabeGadaaakeaaiiGacqWFdpWCdaqhaaWcbaGaemyzaugabaGaeGOmaidaaaaa@30E8@

The two models are equivalent if Π = 12
 MathType@MTEF@5@5@+=feaafiart1ev1aaatCvAUfKttLearuWrP9MDH5MBPbIqV92AaeXatLxBI9gBaebbnrfifHhDYfgasaacH8akY=wiFfYdH8Gipec8Eeeu0xXdbba9frFj0=OqFfea0dXdd9vqai=hGuQ8kuc9pgc9s8qqaq=dirpe0xb9q8qiLsFr0=vr0=vr0dc8meaabaqaciaacaGaaeqabaqabeGadaaakeaadaWcaaqaaiabigdaXaqaaiabikdaYaaaaaa@2E9E@**ZZ' **because they then result in the same log-likelihood function *L*(*μ*, **V**|**y**) = -n2
 MathType@MTEF@5@5@+=feaafiart1ev1aaatCvAUfKttLearuWrP9MDH5MBPbIqV92AaeXatLxBI9gBaebbnrfifHhDYfgasaacH8akY=wiFfYdH8Gipec8Eeeu0xXdbba9frFj0=OqFfea0dXdd9vqai=hGuQ8kuc9pgc9s8qqaq=dirpe0xb9q8qiLsFr0=vr0=vr0dc8meaabaqaciaacaGaaeqabaqabeGadaaakeaadaWcaaqaaiabd6gaUbqaaiabikdaYaaaaaa@2F13@ln(2*π*) - 12
 MathType@MTEF@5@5@+=feaafiart1ev1aaatCvAUfKttLearuWrP9MDH5MBPbIqV92AaeXatLxBI9gBaebbnrfifHhDYfgasaacH8akY=wiFfYdH8Gipec8Eeeu0xXdbba9frFj0=OqFfea0dXdd9vqai=hGuQ8kuc9pgc9s8qqaq=dirpe0xb9q8qiLsFr0=vr0=vr0dc8meaabaqaciaacaGaaeqabaqabeGadaaakeaadaWcaaqaaiabigdaXaqaaiabikdaYaaaaaa@2E9E@ln|**V**| - 12
 MathType@MTEF@5@5@+=feaafiart1ev1aaatCvAUfKttLearuWrP9MDH5MBPbIqV92AaeXatLxBI9gBaebbnrfifHhDYfgasaacH8akY=wiFfYdH8Gipec8Eeeu0xXdbba9frFj0=OqFfea0dXdd9vqai=hGuQ8kuc9pgc9s8qqaq=dirpe0xb9q8qiLsFr0=vr0=vr0dc8meaabaqaciaacaGaaeqabaqabeGadaaakeaadaWcaaqaaiabigdaXaqaaiabikdaYaaaaaa@2E9E@(*y *- *μ*)' **V**^-1^(*y *- *μ*) and the equivalence follows directly from the weak likelihood principle (see e.g. page 194 in [24]).

**Figure 1:**
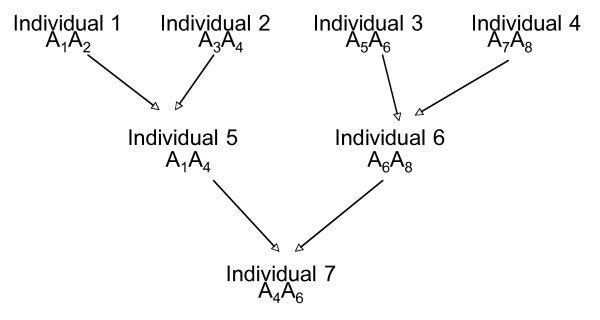
**An example pedigree with a fully informative marker A**. The Z matrix, and corresponding IBD matrix, for this pedigree is given in the text.

The following numerical example shows how **Z **is constructed at a marker position when the marker is fully informative (i.e. all marker alleles are unique), and also that **Π **= 12
 MathType@MTEF@5@5@+=feaafiart1ev1aaatCvAUfKttLearuWrP9MDH5MBPbIqV92AaeXatLxBI9gBaebbnrfifHhDYfgasaacH8akY=wiFfYdH8Gipec8Eeeu0xXdbba9frFj0=OqFfea0dXdd9vqai=hGuQ8kuc9pgc9s8qqaq=dirpe0xb9q8qiLsFr0=vr0=vr0dc8meaabaqaciaacaGaaeqabaqabeGadaaakeaadaWcaaqaaiabigdaXaqaaiabikdaYaaaaaa@2E9E@**ZZ' **Consider the example pedigree in Figure 1 with four individuals in the base generation, two in the second generation and one in the third generation, and a fully informative marker.

The incidence matrix **Z **is obtained by letting the elements of v* be the random effects of the eight independent QTL alleles in the base generation. Let each row in **Z **be indexed according to the labels of the individuals in Figure 1 then:

Z=(11000000001100000000110000000011100100000000010100010100)with v∗=(Q1Q2Q3Q4Q5Q6Q7Q8)
 MathType@MTEF@5@5@+=feaafiart1ev1aaatCvAUfKttLearuWrP9MDH5MBPbIqV92AaeXatLxBI9gBaebbnrfifHhDYfgasaacH8akY=wiFfYdH8Gipec8Eeeu0xXdbba9frFj0=OqFfea0dXdd9vqai=hGuQ8kuc9pgc9s8qqaq=dirpe0xb9q8qiLsFr0=vr0=vr0dc8meaabaqaciaacaGaaeqabaqabeGadaaakeaaieqacqWFAbGwcqGH9aqpdaqadaqaauaabeqahGaaaaaaaeaacqaIXaqmaeaacqaIXaqmaeaacqaIWaamaeaacqaIWaamaeaacqaIWaamaeaacqaIWaamaeaacqaIWaamaeaacqaIWaamaeaacqaIWaamaeaacqaIWaamaeaacqaIXaqmaeaacqaIXaqmaeaacqaIWaamaeaacqaIWaamaeaacqaIWaamaeaacqaIWaamaeaacqaIWaamaeaacqaIWaamaeaacqaIWaamaeaacqaIWaamaeaacqaIXaqmaeaacqaIXaqmaeaacqaIWaamaeaacqaIWaamaeaacqaIWaamaeaacqaIWaamaeaacqaIWaamaeaacqaIWaamaeaacqaIWaamaeaacqaIWaamaeaacqaIXaqmaeaacqaIXaqmaeaacqaIXaqmaeaacqaIWaamaeaacqaIWaamaeaacqaIXaqmaeaacqaIWaamaeaacqaIWaamaeaacqaIWaamaeaacqaIWaamaeaacqaIWaamaeaacqaIWaamaeaacqaIWaamaeaacqaIWaamaeaacqaIWaamaeaacqaIXaqmaeaacqaIWaamaeaacqaIXaqmaeaacqaIWaamaeaacqaIWaamaeaacqaIWaamaeaacqaIXaqmaeaacqaIWaamaeaacqaIXaqmaeaacqaIWaamaeaacqaIWaamaaaacaGLOaGaayzkaaGaee4DaCNaeeyAaKMaeeiDaqNaeeiAaGMaeeiiaaIae8NDayNaey4fIOIaeyypa0ZaaeWaaeaafaqabeacbaaaaaqaaiabdgfarnaaBaaaleaacqaIXaqmaeqaaaGcbaGaemyuae1aaSbaaSqaaiabikdaYaqabaaakeaacqWGrbqudaWgaaWcbaGaeG4mamdabeaaaOqaaiabdgfarnaaBaaaleaacqaI0aanaeqaaaGcbaGaemyuae1aaSbaaSqaaiabiwda1aqabaaakeaacqWGrbqudaWgaaWcbaGaeGOnaydabeaaaOqaaiabdgfarnaaBaaaleaacqaI3aWnaeqaaaGcbaGaemyuae1aaSbaaSqaaiabiIda4aqabaaaaaGccaGLOaGaayzkaaaaaa@831C@

For the pedigree in Figure 1 the corresponding IBD matrix (indexed according to the labels in the figure) is:

Π=(10001200010012012001001212000101201212001012001212011201212012121)with v=(v1v2v3v4v5v6v7)=(Q1+Q2Q3+Q4Q5+Q6Q7+Q8Q1+Q4Q6+Q8Q4+Q6)
 MathType@MTEF@5@5@+=feaafiart1ev1aaatCvAUfKttLearuWrP9MDH5MBPbIqV92AaeXatLxBI9gBaebbnrfifHhDYfgasaacH8akY=wiFfYdH8Gipec8Eeeu0xXdbba9frFj0=OqFfea0dXdd9vqai=hGuQ8kuc9pgc9s8qqaq=dirpe0xb9q8qiLsFr0=vr0=vr0dc8meaabaqaciaacaGaaeqabaqabeGadaaakeaacqqHGoaucqGH9aqpdaqadaqaauaabeqahCaaaaaabaGaeGymaedabaGaeGimaadabaGaeGimaadabaGaeGimaadabaWaaSaaaeaacqaIXaqmaeaacqaIYaGmaaaabaGaeGimaadabaGaeGimaadabaGaeGimaadabaGaeGymaedabaGaeGimaadabaGaeGimaadabaWaaSaaaeaacqaIXaqmaeaacqaIYaGmaaaabaGaeGimaadabaWaaSaaaeaacqaIXaqmaeaacqaIYaGmaaaabaGaeGimaadabaGaeGimaadabaGaeGymaedabaGaeGimaadabaGaeGimaadabaWaaSaaaeaacqaIXaqmaeaacqaIYaGmaaaabaWaaSaaaeaacqaIXaqmaeaacqaIYaGmaaaabaGaeGimaadabaGaeGimaadabaGaeGimaadabaGaeGymaedabaGaeGimaadabaWaaSaaaeaacqaIXaqmaeaacqaIYaGmaaaabaGaeGimaadabaWaaSaaaeaacqaIXaqmaeaacqaIYaGmaaaabaWaaSaaaeaacqaIXaqmaeaacqaIYaGmaaaabaGaeGimaadabaGaeGimaadabaGaeGymaedabaGaeGimaadabaWaaSaaaeaacqaIXaqmaeaacqaIYaGmaaaabaGaeGimaadabaGaeGimaadabaWaaSaaaeaacqaIXaqmaeaacqaIYaGmaaaabaWaaSaaaeaacqaIXaqmaeaacqaIYaGmaaaabaGaeGimaadabaGaeGymaedabaWaaSaaaeaacqaIXaqmaeaacqaIYaGmaaaabaGaeGimaadabaWaaSaaaeaacqaIXaqmaeaacqaIYaGmaaaabaWaaSaaaeaacqaIXaqmaeaacqaIYaGmaaaabaGaeGimaadabaWaaSaaaeaacqaIXaqmaeaacqaIYaGmaaaabaWaaSaaaeaacqaIXaqmaeaacqaIYaGmaaaabaGaeGymaedaaaGaayjkaiaawMcaaiabbEha3jabbMgaPjabbsha0jabbIgaOjabbccaGGqabiab=zha2jabg2da9maabmaabaqbaeqabCqaaaaabaGaemODay3aaSbaaSqaaiabigdaXaqabaaakeaacqWG2bGDdaWgaaWcbaGaeGOmaidabeaaaOqaaiabdAha2naaBaaaleaacqaIZaWmaeqaaaGcbaGaemODay3aaSbaaSqaaiabisda0aqabaaakeaacqWG2bGDdaWgaaWcbaGaeGynaudabeaaaOqaaiabdAha2naaBaaaleaacqaI2aGnaeqaaaGcbaGaemODay3aaSbaaSqaaiabiEda3aqabaaaaaGccaGLOaGaayzkaaGaeyypa0ZaaeWaaeaafaqabeWbbaaaaeaacqWGrbqudaWgaaWcbaGaeGymaedabeaakiabgUcaRiabdgfarnaaBaaaleaacqaIYaGmaeqaaaGcbaGaemyuae1aaSbaaSqaaiabiodaZaqabaGccqGHRaWkcqWGrbqudaWgaaWcbaGaeGinaqdabeaaaOqaaiabdgfarnaaBaaaleaacqaI1aqnaeqaaOGaey4kaSIaemyuae1aaSbaaSqaaiabiAda2aqabaaakeaacqWGrbqudaWgaaWcbaGaeG4naCdabeaakiabgUcaRiabdgfarnaaBaaaleaacqaI4aaoaeqaaaGcbaGaemyuae1aaSbaaSqaaiabigdaXaqabaGccqGHRaWkcqWGrbqudaWgaaWcbaGaeGinaqdabeaaaOqaaiabdgfarnaaBaaaleaacqaI2aGnaeqaaOGaey4kaSIaemyuae1aaSbaaSqaaiabiIda4aqabaaakeaacqWGrbqudaWgaaWcbaGaeGinaqdabeaakiabgUcaRiabdgfarnaaBaaaleaacqaI2aGnaeqaaaaaaOGaayjkaiaawMcaaaaa@B567@

Note that the models (1) and (2) are equivalent since 12
 MathType@MTEF@5@5@+=feaafiart1ev1aaatCvAUfKttLearuWrP9MDH5MBPbIqV92AaeXatLxBI9gBaebbnrfifHhDYfgasaacH8akY=wiFfYdH8Gipec8Eeeu0xXdbba9frFj0=OqFfea0dXdd9vqai=hGuQ8kuc9pgc9s8qqaq=dirpe0xb9q8qiLsFr0=vr0=vr0dc8meaabaqaciaacaGaaeqabaqabeGadaaakeaadaWcaaqaaiabigdaXaqaaiabikdaYaaaaaa@2E9E@**ZZ' **= Π. In this example it was assumed that the QTL was completely linked to a fully informative marker, but a QTL can also be modelled at not fully informative markers, as well as non-marker positions, by adding additional levels to v* that account for the unknown sampling of QTL alleles. The theoretical details are given in Methods. A direct consequence of the presented theory is that Π will be positive definite at non-marker positions and that the number of non-zero eigenvalues at a fully informative marker is equal to twice the number of base individuals (i.e. when the number of marker allele types is equal to the number of base generation alleles). Hence, a VC estimation algorithm that assumes that Π can be inverted (which is the case in the computer packages DMU [25] and ASReml [26] for instance) cannot be used to identify QTL at marker positions where the marker is highly informative.

### Including dominance effects

Model (1) can be extended to include dominance effects by adding an additional variance component d* for the dominance effects

**y **= *μ *+ Zv* + Z_*d*_d* + e

where d* ~ MVN(0, σd2
 MathType@MTEF@5@5@+=feaafiart1ev1aaatCvAUfKttLearuWrP9MDH5MBPbIqV92AaeXatLxBI9gBaebbnrfifHhDYfgasaacH8akY=wiFfYdH8Gipec8Eeeu0xXdbba9frFj0=OqFfea0dXdd9vqai=hGuQ8kuc9pgc9s8qqaq=dirpe0xb9q8qiLsFr0=vr0=vr0dc8meaabaqaciaacaGaaeqabaqabeGadaaakeaaiiGacqWFdpWCdaqhaaWcbaGaemizaqgabaGaeGOmaidaaaaa@30E6@) and σd2
 MathType@MTEF@5@5@+=feaafiart1ev1aaatCvAUfKttLearuWrP9MDH5MBPbIqV92AaeXatLxBI9gBaebbnrfifHhDYfgasaacH8akY=wiFfYdH8Gipec8Eeeu0xXdbba9frFj0=OqFfea0dXdd9vqai=hGuQ8kuc9pgc9s8qqaq=dirpe0xb9q8qiLsFr0=vr0=vr0dc8meaabaqaciaacaGaaeqabaqabeGadaaakeaaiiGacqWFdpWCdaqhaaWcbaGaemizaqgabaGaeGOmaidaaaaa@30E6@ is the variance of the dominance effects. In this model there is a random effect for each allele combination A_*i *_and A_*j *_i.e. each allele combination has an additional dominance effect *δ*_*ij*_. For the pedigree in Figure 1, the matrix **Z**_*d *_(with rows indexed according to the labels in the figure) is given by:

Zd=(0100000000000000000000000000000000000000000000010000000000000000000000000000000000000000010000000000000000000000000000000000000100000000000000000000000000000000000000000000000000000000000000000000000000000000010000000  00000000000000000001000000100000000)
 MathType@MTEF@5@5@+=feaafiart1ev1aaatCvAUfKttLearuWrP9MDH5MBPbIqV92AaeXatLxBI9gBaebbnrfifHhDYfgasaacH8akY=wiFfYdH8Gipec8Eeeu0xXdbba9frFj0=OqFfea0dXdd9vqai=hGuQ8kuc9pgc9s8qqaq=dirpe0xb9q8qiLsFr0=vr0=vr0dc8meaabaqaciaacaGaaeqabaqabeGadaaakeaaieqacqWFAbGwdaWgaaWcbaGaemizaqgabeaakiabg2da9maabmaabaqbaeqabCWhaaaaaaaaaaaaaeaacqaIWaamaeaacqaIXaqmaeaacqaIWaamaeaacqaIWaamaeaacqaIWaamaeaacqaIWaamaeaacqaIWaamaeaacqaIWaamaeaacqaIWaamaeaacqaIWaamaeaacqaIWaamaeaacqaIWaamaeaacqaIWaamaeaacqaIWaamaeaacqaIWaamaeaacqaIWaamaeaacqaIWaamaeaacqaIWaamaeaacqaIWaamaeaacqaIWaamaeaacqaIWaamaeaacqaIWaamaeaacqaIWaamaeaacqaIWaamaeaacqaIWaamaeaacqaIWaamaeaacqaIWaamaeaacqaIWaamaeaacqaIWaamaeaacqaIWaamaeaacqaIWaamaeaacqaIWaamaeaacqaIWaamaeaacqaIWaamaeaacqaIWaamaeaacqaIWaamaeaacqaIWaamaeaacqaIWaamaeaacqaIWaamaeaacqaIWaamaeaacqaIWaamaeaacqaIWaamaeaacqaIWaamaeaacqaIWaamaeaacqaIWaamaeaacqaIWaamaeaacqaIWaamaeaacqaIXaqmaeaacqaIWaamaeaacqaIWaamaeaacqaIWaamaeaacqaIWaamaeaacqaIWaamaeaacqaIWaamaeaacqaIWaamaeaacqaIWaamaeaacqaIWaamaeaacqaIWaamaeaacqaIWaamaeaacqaIWaamaeaacqaIWaamaeaacqaIWaamaeaacqaIWaamaeaacqaIWaamaeaacqaIWaamaeaacqaIWaamaeaacqaIWaamaeaacqaIWaamaeaacqaIWaamaeaacqaIWaamaeaacqaIWaamaeaacqaIWaamaeaacqaIWaamaeaacqaIWaamaeaacqaIWaamaeaacqaIWaamaeaacqaIWaamaeaacqaIWaamaeaacqaIWaamaeaacqaIWaamaeaacqaIWaamaeaacqaIWaamaeaacqaIWaamaeaacqaIWaamaeaacqaIWaamaeaacqaIWaamaeaacqaIWaamaeaacqaIWaamaeaacqaIWaamaeaacqaIXaqmaeaacqaIWaamaeaacqaIWaamaeaacqaIWaamaeaacqaIWaamaeaacqaIWaamaeaacqaIWaamaeaacqaIWaamaeaacqaIWaamaeaacqaIWaamaeaacqaIWaamaeaacqaIWaamaeaacqaIWaamaeaacqaIWaamaeaacqaIWaamaeaacqaIWaamaeaacqaIWaamaeaacqaIWaamaeaacqaIWaamaeaacqaIWaamaeaacqaIWaamaeaacqaIWaamaeaacqaIWaamaeaacqaIWaamaeaacqaIWaamaeaacqaIWaamaeaacqaIWaamaeaacqaIWaamaeaacqaIWaamaeaacqaIWaamaeaacqaIWaamaeaacqaIWaamaeaacqaIWaamaeaacqaIWaamaeaacqaIWaamaeaacqaIWaamaeaacqaIWaamaeaacqaIWaamaeaacqaIXaqmaeaacqaIWaamaeaacqaIWaamaeaacqaIWaamaeaacqaIWaamaeaacqaIWaamaeaacqaIWaamaeaacqaIWaamaeaacqaIWaamaeaacqaIWaamaeaacqaIWaamaeaacqaIWaamaeaacqaIWaamaeaacqaIWaamaeaacqaIWaamaeaacqaIWaamaeaacqaIWaamaeaacqaIWaamaeaacqaIWaamaeaacqaIWaamaeaacqaIWaamaeaacqaIWaamaeaacqaIWaamaeaacqaIWaamaeaacqaIWaamaeaacqaIWaamaeaacqaIWaamaeaacqaIWaamaeaacqaIWaamaeaacqaIWaamaeaacqaIWaamaeaacqaIWaamaeaacqaIWaamaeaacqaIWaamaeaacqaIWaamaeaacqaIWaamaeaacqaIWaamaeaacqaIWaamaeaacqaIWaamaeaacqaIWaamaeaacqaIWaamaeaacqaIWaamaeaacqaIWaamaeaacqaIWaamaeaacqaIWaamaeaacqaIWaamaeaacqaIWaamaeaacqaIWaamaeaacqaIWaamaeaacqaIWaamaeaacqaIWaamaeaacqaIWaamaeaacqaIWaamaeaacqaIWaamaeaacqaIWaamaeaacqaIWaamaeaacqaIWaamaeaacqaIWaamaeaacqaIWaamaeaacqaIWaamaeaacqaIWaamaeaacqaIWaamaeaacqaIWaamaeaacqaIWaamaeaacqaIWaamaeaacqaIWaamaeaacqaIWaamaeaacqaIWaamaeaacqaIWaamaeaacqaIWaamaeaacqaIWaamaeaacqaIWaamaeaacqaIWaamaeaacqaIWaamaeaacqaIWaamaeaacqaIWaamaeaacqaIWaamaeaacqaIWaamaeaacqaIWaamaeaacqaIWaamaeaacqaIWaamaeaacqaIWaamaeaacqaIXaqmaeaacqaIWaamaeaacqaIWaamaeaacqaIWaamaeaacqaIWaamaeaacqaIWaamaeaacqaIWaamaeaacqaIWaamaaGaeeiiaaIaeeiiaasbaeqabCqbaaaaaeaacqqGWaamaeaacqqGWaamaeaacqqGWaamaeaacqqGWaamaeaacqqGWaamaeaacqqGWaamaeaacqqGWaamaeaacqqGWaamaeaacqqGWaamaeaacqqGWaamaeaacqqGWaamaeaacqqGWaamaeaacqqGWaamaeaacqqGWaamaeaacqqGWaamaeaacqqGWaamaeaacqqGWaamaeaacqqGWaamaeaacqqGWaamaeaacqqGXaqmaeaacqqGWaamaeaacqqGWaamaeaacqqGWaamaeaacqqGWaamaeaacqqGWaamaeaacqqGWaamaeaacqqGXaqmaeaacqqGWaamaeaacqqGWaamaeaacqqGWaamaeaacqqGWaamaeaacqqGWaamaeaacqqGWaamaeaacqqGWaamaeaacqqGWaamaaaacaGLOaGaayzkaaaaaa@1E32@

where the number of columns in **Z_*d *_**and the length of d* is 36 since there are (9·8)/2 different allele combinations, and the ordering of columns is such that the element corresponding to *δ*_*ij *_is given in column number (*i*-1)*(8-*i*/2) + *j*. Note also that **Z**_*d*_**Z**'*_d_* is equal to the dominance IBD matrix defined by Xu [23].

### A general algorithm for estimating Z

The matrix **Z **estimated with the algorithm outlined here gives 0.5**ZZ' **equal to the IBD matrix obtained from the single point algorithm developed by Wang et al. [27]. A fully detailed description of the algorithm is given in Methods.

The **Z **matrix is obtained in two steps. In the first step, the first *m *columns of the gametic IBD matrix is constructed, where *m *is twice the number of base generation individuals. Let this matrix be denoted **W**. Additional columns with the factors for the sampling terms due to uncertainty are added if the marker is not fully informative. In the second step, **Z **is obtained by adding the rows of **W **pairwise, so that a row in **Z **relates an element in **y **with a pair of QTL alleles from the base generation.

This is a single point algorithm, but the same principle could be used for multi-point estimation if a multi-point estimated gametic IBD is available.

### Identification of base generation allelic effects

QTL mapping methods are in general based on strict assumptions regarding the genetic architecture of the base generation individuals for the mapping pedigree. Methods using fixed effect models generally assume bi-allelic QTL and that the founder lines are assumed to be fixed for alternative QTL alleles (e.g. [28]). In traditional variance component methods, all founders are assumed unrelated and contribute two alleles each with effects drawn from an allelic effect distribution [1]. QTL mapping experiments are often designed to be a first step in a process to fully dissect the genetic architecture of the trait. The ultimate aim could e.g. be to understand the molecular mechanisms underlying different allelic effects or to identify appropriate genetic markers for efficient marker assisted breeding. It is therefore desirable that one, already during the statistical QTL analysis, obtain as thorough understanding of the genetic architecture of the base generation individuals as possible by e.g. estimating their QTL allele effects before the genetic dissection process is continued. The primary parameter of estimation in the VC QTL model is the variance of the QTL effect, but it is also possible to estimate the effects of each element in the random effect vector using BLUP [20] (see also [6,29]).

The incidence matrix notation presented here provides new opportunities to estimate QTL allele effects of the base generation individuals and to separate these effects from the sampling term effects. The benefit of using the incidence matrix based parameterization of the VC model is shown in the results below.

#### Simulation setup

We simulated an F_2 _pedigree resulting from a cross between two phenotypically divergent outbred strains with a basic structure resembling the Jungle fowl × White leghorn chicken pedigree of Kerje *et al*. [30]. The base generation consisted of four individuals; one male and three females. In the two following generations there were 30 F_1 _and 800 F_2 _individuals. A biallelic QTL was simulated and the phenotypes were simulated as a QTL genotype effect plus a residual effect (i.e. with expected mean equal to 0). Each base generation allele was randomly assigned one of the biallelic types with a 0.5 probability for each type. Only the F_2 _individuals were assumed to be phenotyped. 100 replicates were simulated with a fully informative marker. The BLUP of the base generation QTL alleles were calculated from the mixed model equations (described in detail in the Methods section) with **Z **estimated using the algorithm outlined above. Variance component estimation was done using Fisher's scoring algorithm, and the simulation and estimation algorithms (see Appendix) were programmed in R [31].

Two different simulation experiments were performed to evaluate the possibility of identifying biallelic effects from the BLUP obtained from model (1) and to evaluate the possibility of identifying if a marker homozygote base individual is QTL heterozygous.

The parameters used in the first simulation experiment are given in Table 1. Three different additive biallelic effects were studied and one case with dominance was also included. For each replicate, the BLUP were partitioned into two clusters by minimizing the variance within clusters using the *pam *function in the cluster package of R [31,32]. The correspondence between the clustering and the generated biallelic QTL types in the base generation was subsequently analyzed by summing the number of alleles that had been incorrectly clustered.

**Table 1:** Setup for the simulations with a fully informative marker. 800 F_2 _individuals were simulated with phenotype equal to the sum of the additive QTL effect, dominance QTL effect and residual effect

Percentage of the phenotypic variance explained by the QTL^a^	Additive effect (*a*)^b^	Dominance effect (*d*)	Residual variance
5% additive QTL	3.162	0	95
10% additive QTL	4.472	0	90
20% additive QTL	6.324	0	80
20% add. QTL and 10% dominance	6.324	3.162	70

^a ^Expected additive QTL variance σv2
 MathType@MTEF@5@5@+=feaafiart1ev1aaatCvAUfKttLearuWrP9MDH5MBPbIqV92AaeXatLxBI9gBaebbnrfifHhDYfgasaacH8akY=wiFfYdH8Gipec8Eeeu0xXdbba9frFj0=OqFfea0dXdd9vqai=hGuQ8kuc9pgc9s8qqaq=dirpe0xb9q8qiLsFr0=vr0=vr0dc8meaabaqaciaacaGaaeqabaqabeGadaaakeaaiiGacqWFdpWCdaqhaaWcbaGaemODayhabaGaeGOmaidaaaaa@310A@ equal to 0.5*a*^2^

^b ^The two alternative homozygous genotypes had additive effect *a *and -*a *in the simulated data

In the second set of simulation experiments a 5%, 10% and 20% additive QTL was simulated in the same way as described in Table 1, but **Z **was constructed with the base generation male being marker homozygous.

#### Simulation results

Our method based on the infinite alleles model is powerful in clustering biallelic effects even at moderate effect sizes and the power increases when the proportion of the total variance explained by the QTL increases (Table 2). In the case where dominance was included, only 13% of the dominance BLUP were correctly clustered. The clustering of these effects, however, can be deduced from the clustering of the main effects, because in the biallelic model the dominance effect is added only to the genotypes of the heterozygotes. Hence, it is possible to cluster the biallelic dominance effect accurately as well by identifying the QTL heterozygotes from the estimates of the main QTL effect.

**Table 2:** Simulation results for clustering of biallelic effects using our presentation (eq. (1)) of the infinite alleles model when markers are fully informative. 100 replicates were simulated for each QTL effect

Percentage of the phenotypic variance explained by the QTL	Proportion correctly clustered allele effects	Cluster difference^a^	Estimated allelic QTL variance^b ^(12σv2 MathType@MTEF@5@5@+=feaafiart1ev1aaatCvAUfKttLearuWrP9MDH5MBPbIqV92AaeXatLxBI9gBaebbnrfifHhDYfgasaacH8akY=wiFfYdH8Gipec8Eeeu0xXdbba9frFj0=OqFfea0dXdd9vqai=hGuQ8kuc9pgc9s8qqaq=dirpe0xb9q8qiLsFr0=vr0=vr0dc8meaabaqaciaacaGaaeqabaqabeGadaaakeaadaWcaaqaaiabigdaXaqaaiabikdaYaaaiiGacqWFdpWCdaqhaaWcbaGaemODayhabaGaeGOmaidaaaaa@32FC@)	Estimated dominance variance (σd2 MathType@MTEF@5@5@+=feaafiart1ev1aaatCvAUfKttLearuWrP9MDH5MBPbIqV92AaeXatLxBI9gBaebbnrfifHhDYfgasaacH8akY=wiFfYdH8Gipec8Eeeu0xXdbba9frFj0=OqFfea0dXdd9vqai=hGuQ8kuc9pgc9s8qqaq=dirpe0xb9q8qiLsFr0=vr0=vr0dc8meaabaqaciaacaGaaeqabaqabeGadaaakeaaiiGacqWFdpWCdaqhaaWcbaGaemizaqgabaGaeGOmaidaaaaa@30E6@)	Estimated residual variance
5% additive QTL	0.79	2.63 (0.81)	2.65 (1.16)	-	95.11 (4.85)
10% additive QTL	0.93	3.93 (0.73)	4.99 (1.58)	-	89.85 (4.97)
20% additive QTL	0.97	5.91 (0.65)	9.89 (2.44)	-	78.98 (3.87)
20% add. and 10% dom	0.99	5.39 (1.85)	9.61 (4.46)	10.98 (3.1)	70.43 (2.56)

^a^ Average differences between cluster means. Standard deviations within parentheses

^b ^The allelic variance is estimated from model (1). The expected mean is half the proportion of the total variance explained by the QTL.

In the second set of simulations, the male in the base generation was marker homozygous. Depending on the genetic constitution of this male there will be a bias in the estimated QTL variance. If the male is a QTL heterozygote the QTL variance will be over estimated, and if it is a homozygote it will be underestimated (Table 3). The estimate is, however, consistent if there is a 50% chance of this male being a QTL heterozygote. The reason for the QTL variance being underestimated when the male is homozygous is that the true sampling terms are all zero and their variance will not be proportional to the QTL variance [14]. Since the estimated sampling terms for a QTL homozygote will be close to zero, and those for a heterozygote will differ from zero, it is possible to distinguish between base generation homozygotes and heterozygotes.

**Table 3:** Variance component estimates^a ^from 100 replicates with a marker homozygous individual in the base generation. The marker homozygote had a 50% chance of being QTL heterozygous.

	Allelic QTL variance^b ^(12σv2 MathType@MTEF@5@5@+=feaafiart1ev1aaatCvAUfKttLearuWrP9MDH5MBPbIqV92AaeXatLxBI9gBaebbnrfifHhDYfgasaacH8akY=wiFfYdH8Gipec8Eeeu0xXdbba9frFj0=OqFfea0dXdd9vqai=hGuQ8kuc9pgc9s8qqaq=dirpe0xb9q8qiLsFr0=vr0=vr0dc8meaabaqaciaacaGaaeqabaqabeGadaaakeaadaWcaaqaaiabigdaXaqaaiabikdaYaaaiiGacqWFdpWCdaqhaaWcbaGaemODayhabaGaeGOmaidaaaaa@32FC@)			Residual variance (σv2 MathType@MTEF@5@5@+=feaafiart1ev1aaatCvAUfKttLearuWrP9MDH5MBPbIqV92AaeXatLxBI9gBaebbnrfifHhDYfgasaacH8akY=wiFfYdH8Gipec8Eeeu0xXdbba9frFj0=OqFfea0dXdd9vqai=hGuQ8kuc9pgc9s8qqaq=dirpe0xb9q8qiLsFr0=vr0=vr0dc8meaabaqaciaacaGaaeqabaqabeGadaaakeaaiiGacqWFdpWCdaqhaaWcbaGaemODayhabaGaeGOmaidaaaaa@310A@)		
	All	Homozygotes	Heterozygotes	All	Homozygotes	Heterozygotes
5% additive QTL	2.90 (1.77)	2.16 (1.03)	3.76 (2.07)	94.42 (4.80)	94.17 (5.21)	94.72 (4.30)
10% additive QTL	5.26 (2.81)	3.63 (1.27)	7.71 (2.71)	89.59 (4.90)	89.68 (4.48)	89.45 (5.53)
20% additive QTL	10.27 (6.38)	5.56 (1.30)	17.32 (3.98)	79.67 (4.19)	79.59 (4.42)	79.80 (3.85)

The variance estimates are given as the mean of estimates from all simulations (All), and also as the mean of the estimates divided into two cases: marker homozygote simulated as QTL homozygous (Homozygotes) and marker homozygote simulated as QTL heterozygous (Heterozygotes)

^a ^Standard deviations within parentheses.

^b ^The allelic variance is estimated from model (1). The expected mean is half the proportion of the total variance explained by the QTL

In the simulated pedigree, there were four F_0 _and 40 F_1 _individuals and consequently there are 8 levels in the vector of random effects (v*) corresponding to the base alleles and 40 levels due to sampling terms. The ratio between the variance of the 40 sampling term BLUP and the variance of the 8 base allele BLUP is expected to be close to zero if the base male is a homozygote and it is expected to be greater than one if it is a heterozygote. This difference was clearly shown in the simulations (Table 4) and increased with the size of the QTL. A threshold of 0.5 for the variance ratio was empirically chosen to cluster the simulations into two groups so that all simulations below this threshold were classified into Group A (QTL homozygotes) and all simulations above the threshold were classified into Group B (QTL heterozygotes). Using this classification, all homozygotes and heterozygotes were correctly grouped into Group A and B, respectively, when a 20% QTL was simulated (Table 4). For the 10% QTL case, all homozygotes were correctly grouped and over 75% of the heterozygotes were correctly grouped. When a 5% QTL was simulated over 95% of the homozygotes were correctly grouped and nearly 50% of the heterozygotes were correctly grouped. Hence, this method gives good indication of whether the marker homozygote is QTL heterozygous or not and does not require knowledge of the true QTL variance. In the general case, there is strong support for a heterozygote if the QTL variance is high and the estimated variance ratio between allele effects and sampling terms is greater than one.

**Table 4:** Quantiles for the variance ratios between sampling term BLUP and base allele BLUP split into the two cases where the marker homozygote was either simulated as QTL homozygous or QTL heterozygous

		Min.	5%	25%	50%	75%	95%	Max.
5% additive QTL	Homozygotes	0.05	0.07	0.13	0.17	0.25	0.39	0.67
	Heterozygotes	0.18	0.21	0.34	0.54	0.92	2.09	2.58
10% additive QTL	Homozygotes	0.08	0.10	0.12	0.15	0.21	0.37	0.40
	Heterozygotes	0.26	0.30	0.81	1.19	1.83	2.74	7.06
20% additive QTL	Homozygotes	0.08	0.09	0.12	0.15	0.19	0.34	0.41
	Heterozygotes	0.63	0.94	1.44	1.70	2.29	3.93	9.73

#### Analysis of a QTL in a wild – domestic chicken cross

In a previous standard QTL analysis [28], a QTL on Chicken chromosome 1 [30] was shown to explain 11% of the variance for body weight at 200 days of age, and subsequent analysis of the segregation patterns of the QTL within individual F_1 _sires indicated segregation of the QTL within the founder breeds. This QTL was close (12 cM) to a completely informative marker (LEI246). We calculated the BLUP for the base generation allele effects and subsequently clustered these into two groups assuming a bi-allelic QTL. The results showed that all Jungle fowl have a common allele and all Leghorn hens have common alleles except for one hen, which has one red Jungle fowl allele. Hence, we could identify which base individual that had an allele not fixed within lines. The difference between cluster means was 49.3 g. The estimated fixed effects and variance components were: mean = 1080.3 g, sex effect = 409.2 g, QTL genotype variance = 3251.8, and residual variance = 26028.9.

## Discussion

We have developed a presentation of the VC QTL model where the random effect vector is given in terms of independent levels. Three main points can be concluded from the analytical description of the model and the results presented in the paper: more information can be extracted from QTL analysis by using our alternative model presentation, an improved understanding of VC estimation can be achieved, and the presentation of the model in terms of base allele effects should help to develop new models specifically designed for different structures of dependencies between base generation alleles.

### Estimation of BLUP for base generation allelic effects and sampling term effects

Using our presentation of the infinite alleles model, we could accurately cluster a bi-allelic QTL when markers are fully informative. Furthermore, we were also able develop a measure of whether a marker homozygote is a QTL heterozygote or not, which can be used in both outbred populations and experimental crosses. To our knowledge these developments are novel, but the idea that the sampling variance should be different between the QTL heterozygote and homozygote individuals was mentioned already by Goddard [[14]; p.121]: "If the inheritance of an allele cannot be followed with certainty from parent to offspring [in the infinite alleles model], then a new allele is assumed with effect *pg*_1 _+ (1-*p*)*g*_2 _+ *e *where *g*_1 _and *g*_2 _are the effects of the parental alleles, *p *is the probability that allele 1 was inherited and *e *is a random effect with mean zero and variance equal to *p*(1-*p*) *v*(*g*). If there are only two alleles at this gene then the correct assumption would be that the offspring inherits *g*_1 _with probability *p *and *g*_2 _with probability 1-*p*. On average this implies the same segregation variance as the infinite alleles model but in a particular case, if *g*_1 _and *g*_2 _are similar, then the segregation variance is smaller in the two allele model than in the infinite allele model." Hence, the VC model is capable of giving unbiased estimates for a biallelic QTL [2-5] but this unbiased property of the estimator assumes that a marker homozygote in the base is equally probable of being QTL homozygous or heterozygous. More importantly, the estimate obtained from a single QTL analysis will be severely under- or overestimated depending on whether the marker homozygote is QTL homozygous or heterozygous, respectively.

The infinite alleles model has acquired its name not only because the base generation alleles are assumed to be drawn from an infinite number of different alleles, but also because the sampling terms have previously been included in the allelic effects (as explained in the citation above) and each new sampling term has been thought of as producing a new allelic effect. To us, this production of new QTL alleles through the pedigree due to uncertainty is contra-intuitive and our model notation separates the QTL allele effects from the sampling terms.

The clustering analysis of additive biallelic QTL effects at a fully informative marker presented in the results could have been performed based on the neat transformation of individual genotypic BLUP to individual allele BLUP developed by Nagamine et al. [33,34]. However, these allelic BLUP are calculated for each individual in the pedigree and does not give separate BLUP for the sampling terms, because each allelic effect in this model is a mixture of the base generation allele effects and the sampling terms. Our notation was also easy to extend so that dominance effects could be incorporated, which Nagamine et al. [33] did not attempt in their calculations of allelic BLUP. Furthermore, the transformation given by Nagamine et al. assumes that the IBD matrix is positive definite, which is not the case in marker positions.

### Implications for VC estimation

Our decomposition of the IBD matrix into a low rank incidence matrix shows that IBD matrices will be singular in marker positions and the rank of the IBD matrix will depend on the size of the base generation and the informativeness of the marker. Furthermore, for positions close to markers the IBD matrix will be close to singular. The current VC estimation programmes that are used in human genetics (e.g. SOLAR [35] and MERLIN [36]) have not been developed to deal with large family pedigrees, because so far only small or moderately sized pedigrees have been used, but the need of obtaining fast algorithms for large family pedigrees will be a major task within the near future [37]. VC estimation programmes developed for animal breeding applications [25,26], however, have primarily been developed for estimating the variance of polygenic effects in large pedigrees. The covariance matrix of polygenic effects (i.e. the additive relationship matrix) is positive definite and the VC estimation algorithms that have been developed to estimate polygenic variances have therefore been optimized for situations with non-singular covariance structures, and are unable to handle those cases in VC QTL analysis where the covariance structure is singular (e.g. at marker locations). A way to get around this problem is to add a small positive value to the diagonal of the IBD matrix, which may lead to an extremely ill conditioned optimization problem since the IBD matrix will then be close to singular (i.e. with many eigenvalues close to zero). An alternative is to invert the variance of the response vector directly [38] but neither of these strategies take advantage of the fact that the IBD matrix is lower ranked or close to lower ranked when markers are dense (which was shown in the Methods section).

### Modeling of dependencies between allelic effects in the base generation

We describe the VC QTL model in terms of independent random effects (base generation allele effects and sampling terms) instead of correlated random effects (as in the IBD version of the model). As a consequence it is easier to model correlation structures between base generation alleles, which has been an important issue in linkage-disequilibrium linkage mapping over the past decade [39] where the correlation structure is estimated from marker haplotypes. This method incorporates linkage disequilibrium in the base generation and gives greater power to position a QTL in fine mapping. It can also account for the possibility that marker homozygotes are more likely to be QTL homozygotes. Recently, we have shown [40] that VC modeling can be a powerful tool to identify within line variation in divergent intercrosses by including a correlation structure between the base generation allele effects that is estimated directly in the VC model. An important future development would be to combine these two models so that both the linkage disequilibrium information and the within line variation can be modeled simultaneously. This development, and other possible developments of the VC models where base generation correlation structures are included, should be enhanced by the model presentation given in this paper.

## Conclusion

By defining VC QTL model in terms of independent levels of the random QTL effect, we have developed a simple presentation of the VC model for identification of base generation allele effects in QTL linkage analysis. This development clarifies the definition of the random QTL effect and will be helpful in applications where the aim is to trace allelic QTL effects through a pedigree. Our clarifications of the VC QTL model should also enhance the development of genome scan methods.

## Methods

### Adding uncertainty to the incidence matrix based VC QTL model

A QTL can be modelled at not fully informative markers, as well as non-marker positions, by adding additional levels to v* that account for the sampling of QTL alleles. These sampling terms can be shown to be independent of the QTL allele effects in the base [6,13,14], which motivates the inclusion of these as additional levels in v*. If, for instance, a QTL allele cannot be related to a base generation allele with absolute certainty but it is known that it is one of two alleles with equal probability, then the effect (*Q*_*x*_) of this unknown QTL allele is modelled as:

*Q*_*x *_= 0.5*Q*_1 _+ 0.5*Q*_2 _+ 0.5
 MathType@MTEF@5@5@+=feaafiart1ev1aaatCvAUfKttLearuWrP9MDH5MBPbIqV92AaeXatLxBI9gBaebbnrfifHhDYfgasaacH8akY=wiFfYdH8Gipec8Eeeu0xXdbba9frFj0=OqFfea0dXdd9vqai=hGuQ8kuc9pgc9s8qqaq=dirpe0xb9q8qiLsFr0=vr0=vr0dc8meaabaqaciaacaGaaeqabaqabeGadaaakeaadaGcaaqaaiabicdaWiabc6caUiabiwda1aWcbeaaaaa@2F91@·*ε *    (3)

where *Q*_1 _and *Q*_2 _are the effects of the two alternative QTL alleles and *ε *is the random sampling term. The factor 0.5
 MathType@MTEF@5@5@+=feaafiart1ev1aaatCvAUfKttLearuWrP9MDH5MBPbIqV92AaeXatLxBI9gBaebbnrfifHhDYfgasaacH8akY=wiFfYdH8Gipec8Eeeu0xXdbba9frFj0=OqFfea0dXdd9vqai=hGuQ8kuc9pgc9s8qqaq=dirpe0xb9q8qiLsFr0=vr0=vr0dc8meaabaqaciaacaGaaeqabaqabeGadaaakeaadaGcaaqaaiabicdaWiabc6caUiabiwda1aWcbeaaaaa@2F91@ ensures that the variance of the sampling term *ε *is equal to the variance of random effects in v*.

In the following numerical example the QTL is linked to a marker that is not fully informative. Here, it is not possible to distinguish between the QTL alleles with absolute certainty since the marker is not fully informative. For example, if the marker alleles of individual 1 in Figure 1 were identical, it would not be possible to know which of the two that had been inherited by individual 5. The probability that individual 5 inherited the first of the two alleles is 0.5 and the effect *Q*_*x *_of the unknown allele is either *Q*_1 _or *Q*_2_. This uncertainty is incorporated into the VC model in two steps. In this example, the first step is to change the first and second elements on the fifth row in **Z **to 0.5. This would then be interpreted as *Q*_*x *_= 0.5*Q*_1 _+ 0.5*Q*_2_, but since *Q*_*x *_is either *Q*_1 _or *Q*_2 _a sampling term must be added. This is achieved by applying equation (3): *Q*_*x *_= 0.5*Q*_1 _+ 0.5*Q*_2 _+ 0.5
 MathType@MTEF@5@5@+=feaafiart1ev1aaatCvAUfKttLearuWrP9MDH5MBPbIqV92AaeXatLxBI9gBaebbnrfifHhDYfgasaacH8akY=wiFfYdH8Gipec8Eeeu0xXdbba9frFj0=OqFfea0dXdd9vqai=hGuQ8kuc9pgc9s8qqaq=dirpe0xb9q8qiLsFr0=vr0=vr0dc8meaabaqaciaacaGaaeqabaqabeGadaaakeaadaGcaaqaaiabicdaWiabc6caUiabiwda1aWcbeaaaaa@2F91@·*ε*. The second step is therefore to extend v* to include a ninth element *ε *and add a ninth column to **Z **with element 0.5
 MathType@MTEF@5@5@+=feaafiart1ev1aaatCvAUfKttLearuWrP9MDH5MBPbIqV92AaeXatLxBI9gBaebbnrfifHhDYfgasaacH8akY=wiFfYdH8Gipec8Eeeu0xXdbba9frFj0=OqFfea0dXdd9vqai=hGuQ8kuc9pgc9s8qqaq=dirpe0xb9q8qiLsFr0=vr0=vr0dc8meaabaqaciaacaGaaeqabaqabeGadaaakeaadaGcaaqaaiabicdaWiabc6caUiabiwda1aWcbeaaaaa@2F91@ in the fifth row.

Z=(1100000000011000000000110000000001100.50.50100000.5000001010000101000)v∗=(Q1Q2Q3Q4Q5Q6Q7Q8ε)
 MathType@MTEF@5@5@+=feaafiart1ev1aaatCvAUfKttLearuWrP9MDH5MBPbIqV92AaeXatLxBI9gBaebbnrfifHhDYfgasaacH8akY=wiFfYdH8Gipec8Eeeu0xXdbba9frFj0=OqFfea0dXdd9vqai=hGuQ8kuc9pgc9s8qqaq=dirpe0xb9q8qiLsFr0=vr0=vr0dc8meaabaqaciaacaGaaeqabaqabeGadaaakeaafaqabeqacaaabaacbeGae8NwaOLaeyypa0ZaaeWaaeaafaqabeWbjaaaaaqabaGaeGymaedabaGaeGymaedabaGaeGimaadabaGaeGimaadabaGaeGimaadabaGaeGimaadabaGaeGimaadabaGaeGimaadabaGaeGimaadabaGaeGimaadabaGaeGimaadabaGaeGymaedabaGaeGymaedabaGaeGimaadabaGaeGimaadabaGaeGimaadabaGaeGimaadabaGaeGimaadabaGaeGimaadabaGaeGimaadabaGaeGimaadabaGaeGimaadabaGaeGymaedabaGaeGymaedabaGaeGimaadabaGaeGimaadabaGaeGimaadabaGaeGimaadabaGaeGimaadabaGaeGimaadabaGaeGimaadabaGaeGimaadabaGaeGimaadabaGaeGymaedabaGaeGymaedabaGaeGimaadabaGaeGimaaJaeiOla4IaeGynaudabaGaeGimaaJaeiOla4IaeGynaudabaGaeGimaadabaGaeGymaedabaGaeGimaadabaGaeGimaadabaGaeGimaadabaGaeGimaadabaWaaOaaaeaacqaIWaamcqGGUaGlcqaI1aqnaSqabaaakeaacqaIWaamaeaacqaIWaamaeaacqaIWaamaeaacqaIWaamaeaacqaIWaamaeaacqaIXaqmaeaacqaIWaamaeaacqaIXaqmaeaacqaIWaamaeaacqaIWaamaeaacqaIWaamaeaacqaIWaamaeaacqaIXaqmaeaacqaIWaamaeaacqaIXaqmaeaacqaIWaamaeaacqaIWaamaeaacqaIWaamaaaacaGLOaGaayzkaaaabaGae8NDayNaey4fIOIaeyypa0ZaaeWaaeaafaqabeqcbaaabaqaaiabdgfarnaaBaaaleaacqaIXaqmaeqaaaGcbaGaemyuae1aaSbaaSqaaiabikdaYaqabaaakeaacqWGrbqudaWgaaWcbaGaeG4mamdabeaaaOqaaiabdgfarnaaBaaaleaacqaI0aanaeqaaaGcbaGaemyuae1aaSbaaSqaaiabiwda1aqabaaakeaacqWGrbqudaWgaaWcbaGaeGOnaydabeaaaOqaaiabdgfarnaaBaaaleaacqaI3aWnaeqaaaGcbaGaemyuae1aaSbaaSqaaiabiIda4aqabaaakeaaiiGacqGF1oqzaaaacaGLOaGaayzkaaaaaaaa@8AC0@

Thus, the sampling term is treated as an additional level in the normally distributed random effect.

Similarly v* and **Z **can also be extended to include further uncertainty in the inheritance of the QTL alleles caused by non-informative markers and QTL-scanning at non-marker positions. The formulation of **Z **above gives a VC model (1) that is equivalent to the IBD matrix based VC model (2) since **Π **= 12
 MathType@MTEF@5@5@+=feaafiart1ev1aaatCvAUfKttLearuWrP9MDH5MBPbIqV92AaeXatLxBI9gBaebbnrfifHhDYfgasaacH8akY=wiFfYdH8Gipec8Eeeu0xXdbba9frFj0=OqFfea0dXdd9vqai=hGuQ8kuc9pgc9s8qqaq=dirpe0xb9q8qiLsFr0=vr0=vr0dc8meaabaqaciaacaGaaeqabaqabeGadaaakeaadaWcaaqaaiabigdaXaqaaiabikdaYaaaaaa@2E9E@**ZZ'**.

Two important facts can be noted from our presentation. First of all, the rank of an IBD matrix at a marker position will depend on the informativeness of the marker and the size of the base generation. Secondly, if markers are dense then the IBD matrices at non-marker positions will be positive definite but many of the eigenvalues will be close to zero.

### A general single-point algorithm of estimating Z

Here we describe a general algorithm for obtaining the incidence matrix in (1). The matrix **Z **estimated with this algorithm gives 0.**5ZZ' **equal to the IBD-matrix Π obtained from the single point algorithm developed by Wang et al [27].

The incidence matrix **Z **is obtained by first constructing a matrix **W **describing the haplotype specific inheritance of QTL alleles from the base generation. There are *f *base generation individuals and a total of *n *individuals in the pedigree.

**W **is a matrix of size 2*n *× 2*n*. An element *l *(for *l *= 1,...,2*f*) in row 2*i*-1 is the probability that the paternally inherited QTL allele is identical to the *l*:th allele in the base generation, and an element *l *(*l *= 1,...,2*f*) in row 2*i *is the corresponding probability for the maternally inherited QTL allele. Thus the first 2*f *columns in **W **are the same as the first 2*f *columns of the gametic IBD matrix (as defined in [27])

For simplicity of notation, let a row *k *of **W **be denoted **W**_*k *_and an element in row *k *and column *l *be denoted **W**_*k*,*l*_. The individuals are numbered *i *= 1,2,...,*n *(with ancestors preceding descendents) and the following subscripts are used:

*h *= 1 for paternal allele and 2 for maternal allele

*j*_*ip *_= id-number of the father of individual *i*

*j*_*im *_= id-number of the mother of individual *i*

Each row 2*f*+1 to 2*n *is constructed in two steps. The first step is based on the concept of *probability of descent of a gamete *(*PDQ*_*ij*_) as defined in [27]. The *PDQ *matrix of size 2 × 4 gives the probabilities for the two alleles (row *i*) being inherited from the four possible parental alleles (column *j*).

Step 1: Construction of elements 1 to 2*i*-2 in row 2(*i*-1)+*h*

W2(i−1)+h=PDQh,1W2(jip−1)+1+PDQh,2W2(jip−1)+2+PDQh,3W2(jim−1)+PDQh,4W2(jim−1)+2
 MathType@MTEF@5@5@+=feaafiart1ev1aaatCvAUfKttLearuWrP9MDH5MBPbIqV92AaeXatLxBI9gBaebbnrfifHhDYfgasaacH8akY=wiFfYdH8Gipec8Eeeu0xXdbba9frFj0=OqFfea0dXdd9vqai=hGuQ8kuc9pgc9s8qqaq=dirpe0xb9q8qiLsFr0=vr0=vr0dc8meaabaqaciaacaGaaeqabaqabeGadaaakeaaieqacqWFxbWvdaWgaaWcbaGaeGOmaiJaeiikaGIaemyAaKMaeyOeI0IaeGymaeJaeiykaKIaey4kaSIaemiAaGgabeaakiabg2da9iabdcfaqjabdseaejabdgfarnaaBaaaleaacqWGObaAcqGGSaalcqaIXaqmaeqaaOGae83vaC1aaSbaaSqaaiabikdaYiabcIcaOiabdQgaQnaaBaaameaacqWGPbqAcqWGWbaCaeqaaSGaeyOeI0IaeGymaeJaeiykaKIaey4kaSIaeGymaedabeaakiabgUcaRiabdcfaqjabdseaejabdgfarnaaBaaaleaacqWGObaAcqGGSaalcqaIYaGmaeqaaOGae83vaC1aaSbaaSqaaiabikdaYiabcIcaOiabdQgaQnaaBaaameaacqWGPbqAcqWGWbaCaeqaaSGaeyOeI0IaeGymaeJaeiykaKIaey4kaSIaeGOmaidabeaakiabgUcaRiabdcfaqjabdseaejabdgfarnaaBaaaleaacqWGObaAcqGGSaalcqaIZaWmaeqaaOGae83vaC1aaSbaaSqaaiabikdaYiabcIcaOiabdQgaQnaaBaaameaacqWGPbqAcqWGTbqBaeqaaSGaeyOeI0IaeGymaeJaeiykaKcabeaakiabgUcaRiabdcfaqjabdseaejabdgfarnaaBaaaleaacqWGObaAcqGGSaalcqaI0aanaeqaaOGae83vaC1aaSbaaSqaaiabikdaYiabcIcaOiabdQgaQnaaBaaameaacqWGPbqAcqWGTbqBaeqaaSGaeyOeI0IaeGymaeJaeiykaKIaey4kaSIaeGOmaidabeaaaaa@836E@

Step 2: Construction of the 2 × 2 block diagonal elements

Eih=1−∑k(W2(i−1)+h,k)2
 MathType@MTEF@5@5@+=feaafiart1ev1aaatCvAUfKttLearuWrP9MDH5MBPbIqV92AaeXatLxBI9gBaebbnrfifHhDYfgasaacH8akY=wiFfYdH8Gipec8Eeeu0xXdbba9frFj0=OqFfea0dXdd9vqai=hGuQ8kuc9pgc9s8qqaq=dirpe0xb9q8qiLsFr0=vr0=vr0dc8meaabaqaciaacaGaaeqabaqabeGadaaakeaacqWGfbqrdaWgaaWcbaGaemyAaKMaemiAaGgabeaakiabg2da9maakaaabaGaeGymaeJaeyOeI0YaaabuaeaadaqadaqaaGqabiab=DfaxnaaBaaaleaacqaIYaGmcqGGOaakcqWGPbqAcqGHsislcqaIXaqmcqGGPaqkcqGHRaWkcqWGObaAcqGGSaalcqWGRbWAaeqaaaGccaGLOaGaayzkaaWaaWbaaSqabeaacqaIYaGmaaaabaGaem4AaSgabeqdcqGHris5aaWcbeaaaaa@458F@

**W**_2(*i*-1)+*h*,2(*i*-1)+*h *_= *E*_*ih*_

The matrix **W **will then contain columns with only zeros that correspond to the cases where transmission of alleles from parent to offspring is known without uncertainty. When the matrix **W **is completed the incidence matrix is obtained by summing up the rows in **W **pairwise for each individual:

**Z **= **KW**

where **K **= (1,1)⊗**I**, **I **is the identity matrix of size *n *× *n *and ⊗ is the Kronecker product.

### Mixed model equations for the IBD and Z matrix based VC QTL models

The mixed model equations to solve in the incidence matrix notation (1) is [20]:

(X′XX′ZZ′XZ′Z+σe2G−1)(μv∗)=(X′yZ′y)
 MathType@MTEF@5@5@+=feaafiart1ev1aaatCvAUfKttLearuWrP9MDH5MBPbIqV92AaeXatLxBI9gBaebbnrfifHhDYfgasaacH8akY=wiFfYdH8Gipec8Eeeu0xXdbba9frFj0=OqFfea0dXdd9vqai=hGuQ8kuc9pgc9s8qqaq=dirpe0xb9q8qiLsFr0=vr0=vr0dc8meaabaqaciaacaGaaeqabaqabeGadaaakeaadaqadaqaauaabeqaciaaaeaaieqacuWFybawgaqbaiab=Hfaybqaaiqb=HfayzaafaGae8NwaOfabaGaf8NwaOLbauaacqWFybawaeaacuWFAbGwgaqbaiab=PfaAjabgUcaRGGaciab+n8aZnaaDaaaleaacqWGLbqzaeaacqaIYaGmaaGccqWFhbWrdaahaaWcbeqaaiabgkHiTiabigdaXaaaaaaakiaawIcacaGLPaaadaqadaqaauaabeqaceaaaeaaiiWacqqF8oqBaeaacqWF2bGDcqGHxiIkaaaacaGLOaGaayzkaaGaeyypa0ZaaeWaaeaafaqabeGabaaabaGaf8hwaGLbauaacqWF5bqEaeaacuWFAbGwgaqbaiab=Lha5baaaiaawIcacaGLPaaaaaa@4E47@

with **G **= 12σv2
 MathType@MTEF@5@5@+=feaafiart1ev1aaatCvAUfKttLearuWrP9MDH5MBPbIqV92AaeXatLxBI9gBaebbnrfifHhDYfgasaacH8akY=wiFfYdH8Gipec8Eeeu0xXdbba9frFj0=OqFfea0dXdd9vqai=hGuQ8kuc9pgc9s8qqaq=dirpe0xb9q8qiLsFr0=vr0=vr0dc8meaabaqaciaacaGaaeqabaqabeGadaaakeaadaWcaaqaaiabigdaXaqaaiabikdaYaaaiiGacqWFdpWCdaqhaaWcbaGaemODayhabaGaeGOmaidaaaaa@32FC@**I**,

which gives BLUP for v*, i.e. the QTL allele effects of the base generation individuals and the additional sampling term effects.

The mixed model equations to solve in the IBD-matrix notation (2) is:

(X′XX′XI+σe2G−1)(μv)=(X′yy)
 MathType@MTEF@5@5@+=feaafiart1ev1aaatCvAUfKttLearuWrP9MDH5MBPbIqV92AaeXatLxBI9gBaebbnrfifHhDYfgasaacH8akY=wiFfYdH8Gipec8Eeeu0xXdbba9frFj0=OqFfea0dXdd9vqai=hGuQ8kuc9pgc9s8qqaq=dirpe0xb9q8qiLsFr0=vr0=vr0dc8meaabaqaciaacaGaaeqabaqabeGadaaakeaadaqadaqaauaabeqaciaaaeaaieqacuWFybawgaqbaiab=Hfaybqaaiqb=HfayzaafaaabaGae8hwaGfabaGae8xsaKKaey4kaSccciGae43Wdm3aa0baaSqaaiabdwgaLbqaaiabikdaYaaakiab=DeahnaaCaaaleqabaGaeyOeI0IaeGymaedaaaaaaOGaayjkaiaawMcaamaabmaabaqbaeqabiqaaaqaaGGadiab9X7aTbqaaiab=zha2baaaiaawIcacaGLPaaacqGH9aqpdaqadaqaauaabeqaceaaaeaacuWFybawgaqbaiab=Lha5bqaaiab=Lha5baaaiaawIcacaGLPaaaaaa@482E@

with **G **= σv2
 MathType@MTEF@5@5@+=feaafiart1ev1aaatCvAUfKttLearuWrP9MDH5MBPbIqV92AaeXatLxBI9gBaebbnrfifHhDYfgasaacH8akY=wiFfYdH8Gipec8Eeeu0xXdbba9frFj0=OqFfea0dXdd9vqai=hGuQ8kuc9pgc9s8qqaq=dirpe0xb9q8qiLsFr0=vr0=vr0dc8meaabaqaciaacaGaaeqabaqabeGadaaakeaaiiGacqWFdpWCdaqhaaWcbaGaemODayhabaGaeGOmaidaaaaa@310A@Π,

which gives BLUP for **v**, i.e. the QTL genotype effects for all phenotyped individuals. Here **X **is the design matrix for the fixed effects.

## Authors' contributions

OC initiated the study of VC QTL models. LR drafted the manuscript and initiated the idea of the incidence matrix notation. The paper was then developed through continuous discussions between LR and OC. All authors have read and approved the final version of this paper.

## Appendix

### Fisher scoring algorithm for REML estimates in R code

REML<-function(y,X,n_comp,conv_crit,n_maxiter,lambda_start,delta, Z1, Z2=0, print_results=FALSE, step=1) {

######################################################################################

## Responsible programmer: Lars Rönnegård (lars.ronnegard@lcb.uu.se)

## Date 20060925

#This is the Fisher Scoring algorithm for REML

#See e.g. Johnson & Thompson 1995 J. of Dairy Science 78:449–456.

#The function returns the estimated variance components and fixed effects, together with the log-likelihood

#and convergence information.

#INPUT PARAMTERS (Table 5)

**Table 5:** 

#y	Response vector
#X	Design matrix for fixed effects
#n_comp	Number of different random effects in the model (max.=2 in this version)
#conv_crit	Value that the change in loglikelihood should be less than
#n_maxiter	Maximum number of iterations
#lambda_start	Initial ratio of variance components
#delta	Minimum possible value of the variance component

#WORKING VARIABLES AND OUTPUT PARAMETERS (Table 6)

**Table 6:** 

#A	A matrix which stores ZZ' for all variance components
#phi_start	Staring values for the variance components
#M_phi	Matrix with VC estimates at each iteration
#phi	VC estimate from the latest iteration
#DL	Gradient of the restricted log-likelihood
#FS	Fisher's Information matrix
#V	Variance matrix of y
#P	The projection matrix
#llh	Restricted log-likelihood
#beta_hat	Estimates of fixed effects
#conv_test	Binary variable equal to 1 if the algorithm converges within n_maxiter iterations

######################################################################################

print("REML iteration number")

min.error<-10^-8

n_comp1<-n_comp+1

n_rows<-length(y)

A<-matrix(0,(n_comp1*n_rows),n_rows)

Aj<-matrix(0,n_rows,n_rows)

for (i_comp in 1:n_comp) {

   if (i_comp==1) Aj<-Z1%*%t(Z1)

   if (i_comp==2) Aj<-Z2%*%t(Z2)

   A[((i_comp-1)*n_rows+1):(n_rows*i_comp),1:n_rows]<-Aj

   }

A[(n_comp*n_rows+1):(n_comp1*n_rows),1:n_rows]<-diag(rep(1,n_rows))

res_var<-var(y-X%*%solve(t(X)%*%X)%*%t(X)%*%y)

phi_start<-numeric(n_comp1)

for (i in 1:(n_comp1-1)) {

   phi_start[i]<-lambda_start*res_var

   }

phi_start[n_comp1]<-res_var

dimIBD<-min(dim(A))

M_phi<-matrix(0,(n_maxiter+1),n_comp1)

M_phi[1,1:n_comp1]<-phi_start[1:n_comp1]

phi<-numeric(n_comp1)

DL<-numeric(n_comp1)

DL[1:n_comp1]<-conv_crit+1

FS<-matrix(0,n_comp1,n_comp1)

Aj<-matrix(0,dimIBD,dimIBD)

Ak<-matrix(0,dimIBD,dimIBD)

llh.prev<-1+conv_crit

llh<-0

for (i in 1:n_maxiter){

   V<-matrix(0,dimIBD,dimIBD)

   if (abs(llh-llh.prev)>conv_crit){

      phi<-M_phi[i,]

      for (j in 1:n_comp1){

         Aj<-A[((j-1)*dimIBD+1):(j*dimIBD),1:dimIBD]

         V<-V+phi[j]*Aj

      }

      invV<-solve(V)

      temp<-solve(t(X)%*%invV%*%X)

      P<-invV-invV%*%X%*%(temp)%*%t(X)%*%invV

      for (j in 1:n_comp1){

         Aj<-A[((j-1)*dimIBD+1):(j*dimIBD),1:dimIBD]

         DL[j]<-sum(diag(Aj%*%P))-t(y)%*%P%*%Aj%*%P%*%y

         for (k in j:n_comp1) {

            Ak<-A[((k-1)*dimIBD+1):(k*dimIBD),1:dimIBD]

            FS[j,k]<-sum(diag(Aj%*%P%*%Ak%*%P))

            FS[k,j]<-FS[j,k]

         }

      }

      FS.egen<-eigen(FS, only.values=TRUE)

      FS.min<-min(FS.egen$values)

      if (FS.min>min.error) M_phi[(i+1),]<-phi-step*solve(FS)%*%DL

      if (FS.min<=min.error) {

         print("Negative Hessian")

         identitet<-diag(rep((0.5+abs(FS.min)),min(dim(FS))))

         M_phi[(i+1),]<-phi-step*solve(FS+identitet)%*%DL

      }

      egen<-eigen(V, only.values=TRUE)

      if (min(egen$values>0))ldV<-sum(log(egen$values))

      egen2<-eigen(t(X)%*%invV%*%X, only.values=TRUE)

      ldXVX<-sum(log(egen2$values))

      llh.prev<-llh

      if (min(egen$values)>0) llh<-(ldV+ldXVX+t(y)%*%P%*%y)*(-0.5)

      if (min(egen$values)<=0) llh<-llh.prev-1

      #Truncation at zero

      M_phi[(i+1),]<-0.5*(M_phi[(i+1),]+delta+abs(M_phi[(i+1),]-delta))

      conv_val<-abs(llh-llh.prev)

      if (print_results==TRUE) {

         print("--------------------------------")

         print("Iteration:")

         print(i)

         print("Convergence criteria: Change in log-likelihood")

         print(conv_val)

         print("log-likelihood")

         print(llh)

         print("REML estimates of variance components")

         print("Genotype variance [1] and residual variance [2]")

         print(M_phi[(i+1),])

      }

      if (print_results==FALSE) print(paste(" ",i))

   }

}

conv_test<-1

if (abs(llh-llh.prev)>conv_crit) conv_test<-0

beta_hat<-numeric(min(dim(X)))

beta_hat<-solve(t(X)%*%invV%*%X)%*%t(X)%*%invV%*%y

if (print_results==TRUE) {

   print("Estimates of fixed effects")

   print(beta_hat)

}

list(beta_hat=beta_hat,conv_test=conv_test,conv_val=conv_val,phi=phi,phi_iteration=M_phi,llh=llh)

}

### Algorithm for construction of an F_2 _pedigree with a fully informative marker in R code

simple_F2<-function(n0.males,n0.females,n1.males,n1.females,n2.males,n2.females,QTLvar,RESvar) {

######################################################################################

## Responsible programmer: Lars Rönnegård (lars.ronnegard@lcb.uu.se)

## Date 20060925

## This function constructs an F2 pedigree for a fully informative marker.

## The function returns the simulated phenotypes (y) and the incidence matrix (Z)

## and also the true simulated QTL alleles of the base individuals.

## The phenotypes are simulated assuming a biallelic QTL at the marker position

#INPUT PARAMTERS (Table 9)

**Table 7:** 

#n0.males	No. of males in base generation
#n0.females	No. of females in base generation
#n1.males	No. of males in F1 generation
#n1.females	No. of females in F1 generation
#n2.males	No. of males in F1 generation
#n2.females	No. of females in F1 generation
#QTLvar	Genotypic QTL variance
#RESvar	Residual variance

#WORKING VARIABLES AND OUTPUT PARAMETERS (Table 10)

**Table 8:** 

#n0	Total no. of base generation individuals
#n1	Total no. of F1 individuals
#n2	Total no. of F2 individuals
#n	Total no. of individuals in pedigree
#index.mat	Stores indeces of alleles in the F1 generation
#Z	Incidence matrix for the random base allele effects
#v	Random base allele effects
#y	Vector of phenotypes
#e	Residual effects
#base.alleles	Simulated alleles of the base individuals

######################################################################################

n0=n0.males+n0.females

n1=n1.males+n1.females

n2=n2.males+n2.females

n=n0+n1+n2

Z<-matrix(0,n2,(2*n0))

index.mat<-matrix(0,n1,2)

for (i in 1:n1) {

   sire<-i-n0.males*floor(i/n0.males)+1

   sire.allele<-sample(1:2,1)

   dam<-i-n0.females*floor(i/n0.females)+1

   dam=dam+n0.males

   dam.allele<-sample(1:2,1)

   index.mat[i,]<-c(((sire-1)*2+sire.allele),((dam-1)*2+dam.allele))

}

for (i in 1:n2) {

   sire<-sample(1:n1.males,1)

   sire.allele<-sample(1:2,1)

   dam<-sample(1:n1.females,1)

   dam=dam+n1.males

   dam.allele<-sample(1:2,1)

   Z[i,index.mat[sire,sire.allele]]=Z[i,index.mat[sire,sire.allele]]+1

   Z[i,index.mat[dam,dam.allele]]=Z[i,index.mat[dam,dam.allele]]+1

}

base.alleles <-sample(0:1,(2*n0),replace=TRUE)

v<-base.alleles *sqrt(2*QTLvar)

e<-rnorm(n2,0,sqrt(RESvar))

y<-Z%*%v+e

list(y=y,Z=Z,base.alleles= base.alleles)

}
